# Effect of COVID-19 Pandemic on Food Systems and Determinants of Resilience in Indigenous Communities of Jharkhand State, India: A Serial Cross-Sectional Study

**DOI:** 10.3389/fsufs.2022.724321

**Published:** 2022-03-24

**Authors:** Suparna Ghosh-Jerath, Ridhima Kapoor, Ayushi Dhasmana, Archna Singh, Shauna Downs, Selena Ahmed

**Affiliations:** 1 Indian Institute of Public Health-Delhi, Public Health Foundation of India, Gurgaon, India; 2 Department of Biochemistry, All India Institute of Medical Sciences (AIIMS), New Delhi, India; 3 Department of Urban-Global Public Health, Rutgers School of Public Health, Newark, NJ, United States; 4 Department of Health and Human Development, Montana State University, Bozeman, MT, United States

**Keywords:** tribal, COVID-19, food system, resilience, indigenous population, food production, diets, food environment

## Abstract

The COVID-19 pandemic has globally jeopardized food security, with heightened threats for the most vulnerable including smallholder farmers as well as rural, indigenous populations. A serial cross-sectional study was conducted to document effect of COVID-19 pandemic on food environment, agricultural practices, diets and food security, along with potential determinants of food systems resilience, among vulnerable smallholder farmer households in indigenous communities of Santhal, Munda, and Sauria Paharia of Jharkhand state, India. Telephonic household surveys were conducted in two phases i.e., lockdown and unlock phase to assess the impact of the pandemic on their food systems and agricultural practices. Market surveys were conducted during the unlock phase, to understand the impact on local informal markets. Secondary data on state and district level food production and Government food security programs were also reviewed. For data analysis purpose, a conceptual framework was developed which delineated possible pathways of impact of COVID-19 pandemic on food environment, food security and food consumption patterns along with factors that may offer resilience. Our findings revealed adverse effects on food production and access among all three communities, due to restrictions in movement of farm labor and supplies, along with disruptions in food supply chains and other food-related logistics and services associated with the pandemic and mitigation measures. The pandemic significantly impacted the livelihoods and incomes among all three indigenous communities during both lockdown and unlock phases, which were attributed to a reduction in sale of agricultural produce, distress selling at lower prices and reduced opportunity for daily wage laboring. A significant proportion of respondents also experienced changes in dietary intake patterns. Key determinants of resilience were identified; these included accessibility to agricultural inputs like indigenous seeds, labor available at household level due to back migration and access to diverse food environments, specifically the wild food environment. There is a need for programs and interventions to conserve and revitalize the bio-cultural resources available within these vulnerable indigenous communities and build resilient food systems that depend on shorter food supply chains and utilize indigenous knowledge systems and associated resources, thereby supporting healthy, equitable and sustainable food systems for all.

## Introduction

Over 820 million people in the world suffer from hunger, while about two billion people experience moderate or severe food insecurity (The United Nations, 2019). The world is not on track to achieve Sustainable Development Goal (SDG) 2 of Zero Hunger by 2030 and if these trends continue, the number of people affected by hunger would surpass 840 million by 2030 (Kretchmer, 2020). Evidence suggests that manmade conflicts, climate change, and economic downturns can lead to acute hunger among 135 million people globally. The COVID-19 pandemic could double that number, putting millions of people at risk of suffering from acute hunger (Anthem, 2020). In fact, the pandemic has jeopardized food security in communities globally, with heightened threats for the most vulnerable including smallholder farmers as well as rural, and indigenous populations (FAO, 2020b). It is well recognized that sustainable food systems can play a critical role in creating a zero-hunger world (The Economist, 2021). However, the COVID-19 pandemic has put the global food supply system under the most vigorous pressure tests (Vos et al., 2020), calling for a need to strengthen the resilience of food systems to support food security for all (Bén, 2020; Zurayk, 2020).

In order to contain the impact of the COVID-19 pandemic, governments in all the countries, irrespective of their Gross National Income (GNI) per capita, imposed a range of measures (like bans on public gatherings, restrictions on mobility, temporary closure of academic institutions, markets, private, and government organizations and other measures) to prevent the spread of the virus. These collective measures are generally known as a "lockdown," which is a community containment strategy that helps to restrict the contact of unidentified or asymptomatic cases with the community (Manzar et al., 2020). This resulted in cross-cutting implications for all aspects of food systems from production, distribution, and storage to food environments, consumption, and waste, at all levels and scales (Zurayk, 2020). The lockdown measures and accompanying mobility restriction, though crucial to contain the pandemic and minimize loss of life, have created significant economic stresses with adverse consequences for food security and hunger, with populations who are already vulnerable to poverty and malnutrition being disproportionately impacted (Laborde et al., 2020). Among the worst affected are two-thirds of the world's poor comprising of smallholder farmers who depend on agriculture for income as well as for household food supply (Castaneda et al., 2016).

The agriculture sector has typically been exempted from lockdown restrictions in most of the countries to ensure continuity of food production. Although analysis at a macro-level (i.e., global and national) during the initial months of the pandemic indicated that the COVID-19 pandemic did not substantially compromise food availability (Bén, 2020; Devereux et al., 2020), later on, the negative impact on food availability was more evident in different parts of the world (FSIN Global Network Against Food Crises, 2021). Further, studies and data sets have also indicated disruptions of national and global supply chains with impacts of reduced food exports and imports on the global food market (Aday and Aday, 2020; FAO, 2020a). At the local level, multiple studies are emerging that demonstrate how food access was threatened due to supply chain disruptions coupled with other factors such as increased food prices relative to wages and income (Devereux et al., 2020). For example, the pandemic restricted the movement of people and goods as well as disrupted access to farm inputs, labor availability and schedules, transportation, safety, and management practices (Torero, 2020). This led to a cascade along agri-food supply chains, impacting markets, prices, income and livelihoods, food accessibility and security and, nutrition (Muscogiuri et al., 2020; TechnoServe, 2020). These supply chain disruptions linked to the COVID-19 lockdown have been notable among the vulnerable populations such as indigenous smallholder farmers, impacting their food environments and overall food systems (ILO, 2020). Further, the compromised diets consumed by these communities may impact their metabolic health and nutritional status, which may have important implications for the progression and pathology of COVID-19 (Muscogiuri et al., 2020), exacerbating existing health disparities.

The need for resilient and equitable food systems has been re-emphasized in response to the COVID-19 pandemic for supporting food security for all (Bén, 2020), including vulnerable populations with heightened health disparities. A food systems resilience framework addresses the complex relationships within food systems despite disturbances (Ericksen et al., 2009; Ingram et al., 2010; Tendall et al., 2015), such as climate change, habitat destruction, the current COVID-19 pandemic, as well as the interaction of these disturbances (Ahmed et al., 2020). While smallholder farmers including those in indigenous communities are vulnerable to global change including pandemics, they also variably demonstrate unique attributes of resilience concerning their food systems. Some of these smallholder farming communities are geographically positioned in the hard-to-reach and challenging terrains of low- and middle-income countries, (Rapsomanikis, 2015; Haga, 2020) where they are the crucial providers of food in areas with some of the most pressing needs for food access (Bén, 2020). Further, they contribute to national food security, especially at times when trade is compromised. They are wellpositioned to ensure continuity in food supplies amidst complex logistical and transport issues (Haga, 2020; Lopez-Ridaura et al., 2021). The use of family labor by smallholder farms may enable them to overcome possible labor shortages in the context of supply chain disruptions with regard to harvesting, getting food to market, and other farm-related activities (Haga, 2020; Tripathi et al., 2021). Based on the resilience theory, where diversity is a key socio-ecological determinant of resilience (Walker and Salt, 2012), the food systems of smallholder farmers may have more diverse types of food environments. Smallholder farmers in low and middle-income countries (LMICs) often manage wild and cultivated food environments while accessing formal and informal markets (Downs et al., 2020). It is hypothesized that access to a greater number of types of food environments in the context of global change may offer greater resilience toward supporting diets and food security (Ahmed et al., 2020).

India, a country in Southeast Asia, is home to about 120 million smallholder farmers who constitute over 80% of the agricultural sector in India (Ministry of Agriculture Farmers Welfare, 2016). The nationwide lockdown that was announced on 24th March 2020 in India, came at an unfortunate time for farmers, as it coincided with the harvest season for the *Rabi* (winter) crops in many parts of the country (GAIN, 2021). This lockdown led to the closure of multiple government and private establishments and restricted inter and intra-state movements (Hindustan Times, 2020; Kar, 2020; Times of India, 2020) which further added to the challenges around agricultural activities and the supply chain. This compounded the misery of the smallholder farmers who were already burdened by challenges around limited agrotechnology, climate change, price volatility, and rising debts (Ministry of Agriculture Farmers Welfare, 2016). All these changes worsened the living conditions of many smallholder farmers, who faced additional challenges due to loss of livelihoods and stagnant wages (Harris et al., 2020). Further, the closure of Anganwadi centres (maternal and child health centres) and schools, which are the main sites for delivery of government's supplementary feeding programs [like Integrated Child Development Services (ICDS) and Mid-day meal (MDM) programs], had exacerbated the nutritional vulnerability amongst the families of these smallholder farmer communities (Sinha, 2021).

Jharkhand, an eastern Indian state known for its rich biodiverse agroforestry (Kumar and Saikia, 2020), is home to several indigenous communities that constitute 26.2% of the state's population (Census of India, 2011). About 80% of the population in this state's rural areas derive their livelihoods from agriculture (Ministry of Environment Forests Climate Change, 2018). Most of the state population comprises smallholder farmers, with about 50% of them having land ownership of fewer than 0.4 hectares (ha). The indigenous communities of India, recognized by the government as “Scheduled tribes (STs),” are among the most food insecure and nutritionally vulnerable communities (MoHFW Ministry of Tribal Affairs, 2020; UNICEF India, 2020). A majority of these STs comprise marginal and smallholder farmers (Ministry of Tribal Affairs, 2002), who extensively rely on their indigenous local food systems for nutrition and livelihood (Bhattacharjee et al., 2009). During the countrywide lockdown imposed from 24th March to 7th June 2020, the lives of many indigenous communities in Jharkhand were affected, especially for those residing in the hard-to-reach geographies of the state (Indiaspend, 2020). Studies and surveys in Jharkhand have reported impacts on agricultural practices and livestock management as well as disruptions in supply chains and functioning of supplementary feeding programs (NABARD, 2020; State Food Commission Social Audit Unit Jharkhand, 2020; Nair et al., 2021). Estimates have suggested that disruption to nutrition programs could lead to additional cases of underweight and wasting respectively as well as an increase in deaths due to wasting in the state of Jharkhand (Rajpal et al., 2020; Roberton et al., 2020; Bahl et al., 2021).

Exploring the effect of COVID-19 pandemic and lockdown measures on the food environment of vulnerable indigenous communities of Jharkhand as well as identification of determinants of resilience is thus crucial. Hence, the present paper documents the effect of the COVID-19 pandemic on the food environment, agricultural practices, diets, and food security of vulnerable smallholder farmer households in indigenous communities of Santhal, Munda, and Sauria Paharia of Jharkhand, India. This study further explored the potential determinants of resilience with regards to the food systems of these indigenous communities during the COVID-19 pandemic. The findings from this study will provide valuable insights into the status of food systems, food security, and diets of these communities toward supporting future efforts to ensure food security in the context of global change.

## Methods

### Study Area, Population, and Setting

**Figure 1:**
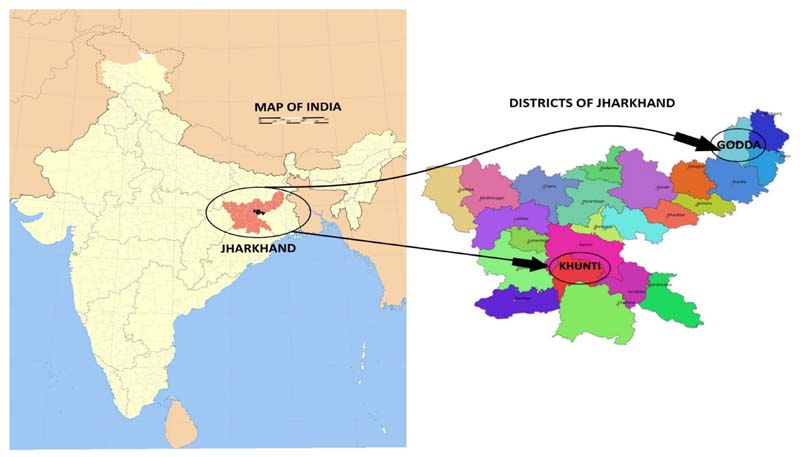
Selection of study districts in Jharkhand.

Three indigenous communities in the Indian state of Jharkhand, namely, Sauria Paharia, Santhal, and Munda communities were included in this study. The Santhal and Munda communities were selected as these are amongst the most populous indigenous communities in Jharkhand (Census of India, 2011). In addition, the Sauria Paharia community was included in this study as it is a particularly vulnerable tribal group (PVTG) owing to its pre-agricultural level of technology, low level of literacy, economic vulnerability, and declining population (Press Information Bureau, 2019). The study population for the Household (HH) survey comprised adult members (18 years of age and above) of HHs residing in 44 study villages located in purposively selected geographically diverse blocks of Sunderpahari, Boarijor, Poreyahat, and Pathargama in Godda, and Murhu and Torpa in Khunti districts respectively (Figure 1). The Godda district is home to Sauria Paharia and Santhal communities, with regions surrounded by undulating uplands, long ridges, and depressions along with scattered hillocks covered with forests (Godda, 2021). The Khunti district, on the other hand, is predominantly populated by the Munda community, and 40% of the district is covered with forests, with both hilly terrain and plain lands, respectively (Singh and Kumar, 2016).

### Study Design and Duration

**Figure 2:**
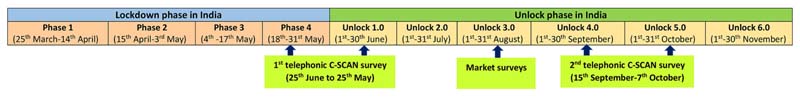
Timeline of data collection period of C-SCAN and market survey.

A serial (repeated) cross-sectional HH survey was conducted telephonically in two phases (Figure 2) to understand and differentiate the impact of the COVID-19 pandemic on food systems at variable levels of restrictions imposed by the national and state governments The first phase of data collection was between 25th May to 25th June 2020. This included both lockdown period and early week of post lockdown phase due to COVID-19 pandemic in the study area and is referred to as *“Lockdown phase.”* During this period, most services, organizations, factories, and markets remained closed and no public gatherings and intra- or inter-state travel were allowed. The lockdown phase coincided with the sowing season of the agricultural calendar in the study area. The second phase of data collection was between 15th September to 7th October 2020 during the fourth phase of the countrywide unlock i.e., ease of lockdown restrictions in India, and is referred to as the *“Unlock phase.”* During this phase, most services, establishments, and factories were allowed to open and relaxations were announced for agricultural businesses, travel within cities, and states and selling of farm supplies in open markets. In addition, market surveys with food vendors were conducted during the third phase of countrywide unlock in August 2020 (Figure 2).

### Study Tools

For assessing the effects of COVID-19 pandemic on food systems and agricultural practices of indigenous communities, HH surveys were conducted using a tool titled *“COVID-19 Surveillance Community Action Network* (C-SCAN)" for food systems, which was developed based on the food environment typology framework (Downs et al., 2020) and has been previously administered in China (Ahmed et al., 2020). The survey consisted of six parts and elicited information regarding the socio-demographic profiles, different types of food sources accessed, and perceptions about food security aspects including food availability, access and utilization, diets consumed, HH income, and farming and gardening systems. The survey aimed to examine how consumers (smallholder farmers) in indigenous communities interacted and utilized various types of food environments when food systems were disrupted due to shocks and stressors including the COVID-19 pandemic and associated lockdown measures. Since the hypotheses that were applied to develop the C-SCAN survey tool delineated factors that may offer resilience in different food systems, the responses were utilized to rapidly gain insights into the resiliency of indigenous food systems. Specifically, understanding the resilience of indigenous food systems in the context of COVID-19 pandemic was achieved in the C-SCAN survey tool through questions eliciting information regarding the traditional ecological knowledge of the communities, access to diverse food environments with predominant reliance on wild and cultivated food sources, access to informal built food environments that rely on locally produced foods, and farming practices that may utilize specific agroecological approaches. The questions in the C-SCAN survey tool were framed in the form of binary Yes/No responses which allowed for rapid assessment and analysis, while selected questions also had provision for further qualitative descriptions. For use in a local context, we translated the survey tool from English to Hindi to facilitate the communication of core team members with the indigenous communities, who mainly understood the Hindi language. In case of the Sauria Paharia community, where people were more comfortable with their native Paharia dialect, the survey was administered by local Sauria Paharia field investigators. For the market survey, a survey tool adapted from Downs et al. (2020) was administered to the food vendors by the local field investigators (who were trained by the core team on administering the survey). The market survey elicited information on the following parameters: (i) the main types of foods sold in the market in terms of food groups, (ii) food prices pre- and post-lockdown, along with perceived reasons for change, (iii) sources of food procurement and any change in procurement patterns with rationale for shifting behavior, and (iv) change in sales of specific food items (during and post lockdown) (along with perceived reasons). Additionally, a set of interview questions were also administered to the food vendors, which elicited their perceptions on post-lockdown changes in sales and income.

### Sample Size Calculation

Using Epi-Info Software, Version 7.2, the sample size was calculated based on a preliminary study conducted by co-authors, which reported a 93% change in income among smallholder farmers in China, due to the COVID-19 pandemic (Ahmed et al., 2020). A sample size of 104 was estimated considering an absolute precision of 6%, a design effect of 1.5, and a 95% confidence interval. To compensate for the 50% non-response rate (considering that the survey was to be conducted telephonically in hard-to-reach rural areas of Jharkhand), a sample size of 150 was arrived at. Given the need for a rapid response to accomplish research in the context of the COVID-19 pandemic and lockdown measures, this sample size was also commensurate with the available resources required to conduct this rapid survey.

### Sampling

The study villages were selected using probability proportional to size sampling as part of a larger study which is exploring the role of indigenous foods on food and nutrition security of indigenous communities of Jharkhand, details of which are reported elsewhere (Ghosh-Jerath et al., 2019). Given the extraordinary circumstances of the COVID-19 pandemic and the need to carry out rapid and continuous assessments of the effects of the pandemic and associated phases of lockdown measures on food environments and agricultural practices, we used a convenience sampling of HHs from the Sauria Paharia, Santhal and Munda communities in the selected villages that were surveyed as part of the larger study (Ghosh-Jerath et al., 2019). We further conveniently prepared a list of HHs that had access to either mobile phones or landline telephones. All HHs from this list (*n* = 946), were approached during the lockdown and unlock phase. Additional HHs in the study villages were also approached through the local field team using a snowball sampling technique (75 in the lockdown and 142 in the unlock phase). Out of all these approached HHs, 152 surveys (Sauria Paharia, *n* = 49; Santhals, *n* = 35, Munda, *n* = 68) in the lockdown phase (response rate: 14.9%) and 151 surveys (Sauria Paharia, *n* = 72; Santhals, *n* = 20, Munda, *n* = 59) in unlock phase (response rate: 13.8%) were telephonically completed (Supplementary Table 1).

For the market surveys, a total of eight local weekly markets (list provided as Supplementary Table 2) that are frequently accessed by Sauria Paharia, Santhal and Munda communities in the selected blocks of Godda and Khunti districts were purposively chosen. Additionally, a vendor survey was conducted with a convenience sample of 56 vendors (6 to 7 vendors per market) in a total of 8 markets. The criteria for the selection of the respondents (HH members and food vendors) was based upon their availability and consent for participation.

### Data Collection and Data Entry

The field investigators administered C-SCAN survey tool telephonically using paper forms. The responses received were entered in the paper forms by the field investigators and shared with the core team. This ensured the necessity of social distancing in the unusual circumstances amidst the COVID-19 pandemic for safety and infection prevention while at the same time reaching out to geographically isolated communities in the state. Survey administration by local Sauria Paharia field investigators helped in efficient rapport building with the communities, thus making it easier for the community to discuss their situation openly without hesitation. For data entry purposes, the C-SCAN survey tool was incorporated in CS-Pro Software, Version 7.3, that provided in-built checks (range, context, and logic checks) for ensuring data quality. The market and vendor surveys were conducted as face-to-face interviews using paper forms by the local field investigators and the data was entered in an excel sheet.

### Data Triangulation With Secondary Literature

In order to supplement our study findings, an online search was conducted to identify state and district level government reports that have documented the impact of COVID-19 pandemic on different aspects of food systems in Jharkhand. For this purpose, many ministry websites were searched. We were able to extract data on two main aspects:

(1) Food production in Jharkhand during the lockdown phase: This data was collected by the National Bank for Agriculture and Rural development (NABARD, 2020) from 29 April 2020 to 04 May 2020, through an online questionnaire that was administered to district development managers, based on their interactions with various stakeholders, viz. farmers, government officials, members of self-help groups, farmer clubs, and farmer producer organizations;
(2) District-level access to Public Distribution System (PDS) and other government food security programs during the lockdown and unlock phase. For information on this, the state website of PDS distribution (Department of Food, Public Distribution and Consumer Affairs, Government of Jharkhand, 2020) was reviewed to document the transaction status of different commodities under PDS during the pre-COVID-19 period, lockdown phase and unlock phase in Godda and Khunti districts. Additionally, a state audit report on the status of two supplementary feeding programs, namely, MDM and ICDS programs during the lockdown phase was also reviewed (State Food Commission Social Audit Unit Jharkhand, 2020).

### Data Analysis

**Figure 3:**
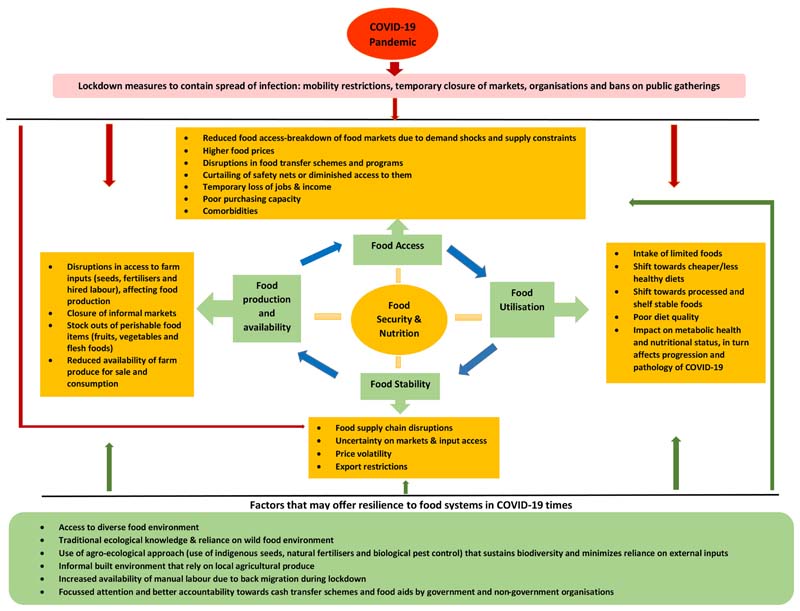
Conceptual framework: Impact of COVID-19 pandemic on food security among vulnerable populations and factors offering resilience.

A conceptual framework (adapted from HLPE, 2020) was developed for the data analysis purpose, which delineated the possible pathways on how the COVID-19 pandemic and the resulting lockdown may have affected the food environment, food security, and food consumption patterns of the vulnerable populations. Further, based on the food environment typology framework (Ahmed et al., 2020; Downs et al., 2020), a set of factors were incorporated in this framework, that were likely to offer resilience to the COVID-19 pandemic's impact on food systems. These factors included access to diverse types of food environments, reliance on wild and cultivated food sources, possession of traditional ecological knowledge, use of the agroecological approach for food production, and access to informal built food environments that rely on local agricultural produce (Figure 3).

The raw data from the C-SCAN tool and market surveys were exported and cleaned in MS Excel, and the analysis was performed in Stata, version 15.1 (StataCorp, 2017). The quantitative variables were analyzed using descriptive statistics, which included computation of frequency counts and percentages for categorical variables, mean, standard deviation, median, and interquartile ranges (IQR) for continuous variables. Inferential statistical analyses were conducted using the chi-square test for comparison of categorical variables and *t*-test for continuous outcomes. Since we wanted to compare the characteristics of the HHs surveyed in lockdown and unlock phases, we used two-tailed tests of hypotheses and a *p*-value < 0.05 as the criteria for statistical significance. The qualitative responses were translated to English and cleaned by removing filler words (and, or, those, etc.) and categorized under specific additional variables (such as type of foods that were easy to access, difficult to access, reasons for change in income, etc.). Based on the conceptual framework (Figure 3), qualitative responses were manually coded according to key sub-theme categories and subsequently organized based on the broader concepts included in the conceptual framework. In order to substantiate the study findings, the responses from our telephonic and market surveys were further triangulated with the district level and state level secondary data.

### Ethical Considerations

Necessary approvals were obtained from the Institutional Ethics Committee of Indian Institute of Public Health- Delhi Public Health Foundation of India to protect human subjects through ensuring the highest ethical standards and conduct in the present research study. Verbal informed consent was obtained from the HH survey respondents and market vendors while providing them with all necessary details regarding the study. Due permissions for recording the telephonic interviews were obtained from the respondents. All the collected data were kept confidential and safe.

## Results

Amongst the surveyed HHs, the majority of the respondents were males (76% in lockdown phase and 72% in unlock phase), with a mean age of 33 ± 11 and 32 ± 9 years, respectively. Most HHs practiced farming on agricultural lands, while some HHs grew food in home gardens (*Baris*). Among the PVTGs, i.e., the Sauria Paharia community, nearly three-fourths of the HHs (during the administration of both surveys) reported farming on burnt patches of forest land (known as *Kurwa* farming). Farming was the primary source of income in majority of the HHs (72% in lockdown phase and 74% in unlock phase), while some HHs (18% in lockdown phase and 17% in unlock phase) were also engaged in daily wage laboring (Table 1).

The following sections discuss the effect of COVID-19 pandemic and the resulting lockdown on various aspects of HH food security, i.e., food availability, access, and utilization in the three indigenous communities during the lockdown, and the unlock phases, supplemented with secondary data on food production and access to government programs. Further, findings from market and vendor surveys are presented to highlight the impact of the COVID-19 pandemic on the informal markets accessed by the three indigenous communities. Finally, findings from both C-SCAN and market surveys are dissected to explore the factors and mechanisms that offered resilience to these indigenous communities during the pandemic.

### Perceived Impacts of COVID-19 Pandemic on Different Aspects of Household Food Security

#### Impact on Food Availability

##### COVID-19 Pandemic and the Food Environment

**Figure 4:**
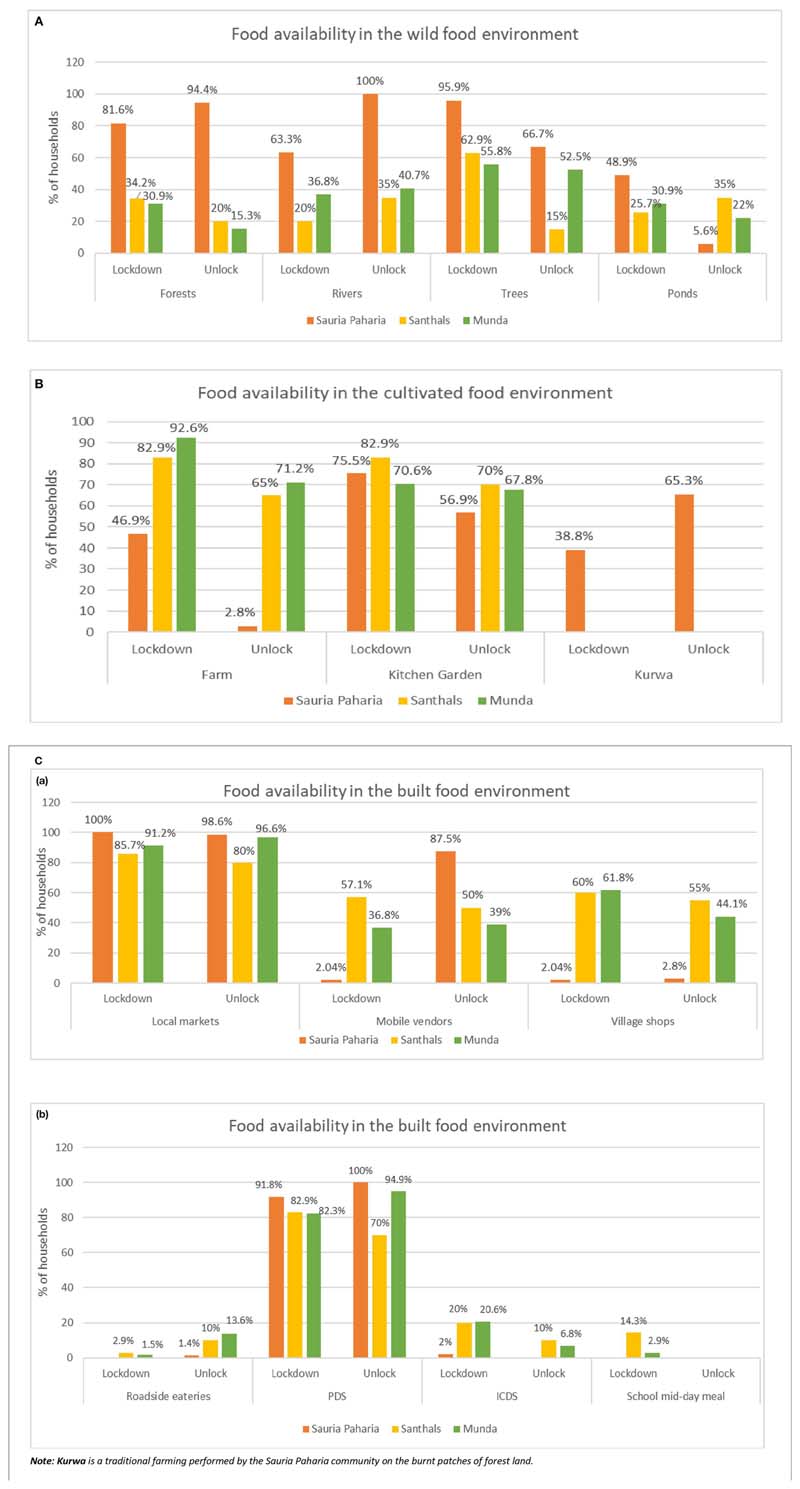
Food environment of the indigenous communities during the COVID-19 pandemic. **(A)** Food availability in the wild food environment during the lockdown phase (May-June, 2020) and the unlock phase (September-October, 2020). **(B)** Food availability in the cultivated food environment during the lockdown phase (May-June, 2020) and the unlock phase (September-October, 2020). **(C)** (a,b) Food availability in the built food environment during the lockdown phase (May-June, 2020) and the unlock phase (September-October, 2020).

During both the lockdown and subsequent unlock phases of the COVID-19 pandemic examined here, the surveyed communities reported accessing foods mainly from their natural food environment. Specifically, they procured food from wild food environments including forests, local water bodies, and surrounding natural vegetation as well as from cultivated food environments including fields, and gardens (Figure 4). The Sauria Paharia HHs reported the highest availability of food from the wild food environment, while the Santhals and Mundas mainly reported food availability from the cultivated food environment. During the lockdown phase, a higher number of Sauria Paharia HHs reported food availability from farms, as most of the community members practiced settled agriculture on plain farmlands (done primarily once a year), at the time of the survey. On the other hand, as the community mainly sourced *Kurwa* lands during the unlock phase, a higher food availability was reported from that source. Nearly all HHs reported food availability in the built food environment including weekly informal markets, mobile vendors, and corner shops within the villages. In addition, a large majority (85.5%) of HHs from all three communities reported availability of subsidized foods in the form of grains, sugar, salt, etc., in the formal markets, which included fair price shops under PDS, a federal food security program in India. Only about one-fifth of the HHs in Santhal and Munda communities reported the availability of supplementary food in the form of take-home ration that was delivered to their door-steps from Anganwadi centers under ICDS. Among Sauria Paharias, only 2% of HHs reported availability of supplementary food from Anganwadi centers. A significant difference (p<0.001) was also observed in the availability of dry ration from MDM program during the two phases: due to the closure of schools, a few Santhal (14%) and Munda (3%) HHs reported receiving additional food in the form of dry rations (like cereals and pulses) through MDM, however the distribution was reportedly discontinued during the unlock phase, even though the schools remained closed.

##### Food Production During the COVID-19 Pandemic

The majority of the HHs (74% in lockdown and 54% in unlock phases) did not report any changes in their crop production and yields. However, disruptions in regular access to farm inputs were reported, notably in Santhal and Munda communities. A substantial number of HHs in both these communities (60−69% in lockdown and 35−47% in unlock phases) reported hardships in procuring farm inputs (seeds and fertilizers), as markets were open for limited durations (even during the unlock phase) and prices were higher owing to shortage of supplies. A few Santhal and Munda HHs also reported hardships in arranging manual labor for agricultural work owing to concerns around the spread of COVID-19 infection. During both the phases, Sauria Paharia HHs were relatively less impacted as they chiefly relied on indigenous seeds and organic manure for agriculture. In addition, about 12% of HHs (*n* = 6/49) in the Sauria Paharia community also reported greater access to foods sourced from wild food environment such as forests, local water bodies, and natural habitats. Due to a marked decline in the procurement of various agronomic inputs during the lockdown phase, a small proportion of HHs (*n* = 5/103) in Santhal and Munda communities reported a delay in the usual sowing time, while no such changes were reported during the unlock phase. In contrast, nearly three-quarters of HHs (72%) in the Sauria Paharia community reported sowing their crops earlier (as compared to pre-COVID-19 times) during the lockdown, as many migrant HH members had returned to their villages, leading to surplus manual labor for the sowing process. Surveyed HHs in Sauria Paharia community reported no changes in farming schedule during the unlock phase (Table 2).

**Figure 5:**
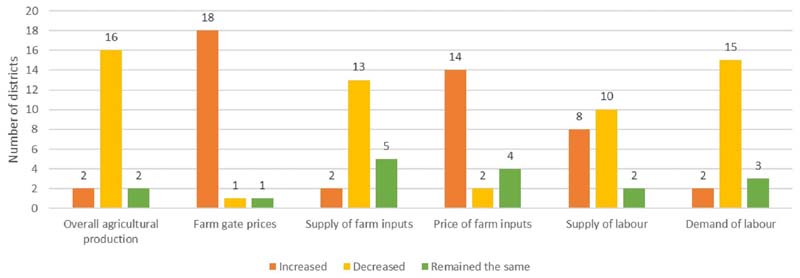
Impact assessment of COVID-19 pandemic on agricultural production in Jharkhand. Source: NABARD (2020).

The secondary data on state-level impact of COVID-19 pandemic on agriculture production in Jharkhand (NABARD, 2020) (Figure 5) reported similar trends. The majority of the districts (16 out of 20 surveyed districts) in Jharkhand experienced a reduction in overall agricultural production, with an average production decrease of 6.7%. However, the farm production was relatively less impacted, as compared to other allied sectors like horticulture, animal husbandry, and fisheries (a production decrease between 9 to 30%). A possible reason could be the completion of crop production and harvesting in many districts before the lockdown announcement. The farm gate prices (i.e., prices of farm produce) were adversely impacted in almost all districts, although the average % decrease (0.8%) was nominal. The main reasons cited were reduced consumer demand due to lack of transport and shutting down of local weekly markets. The lockdown restrictions further resulted in a reduced supply of farm inputs (like seeds, fertilizers, pesticides, machinery, and fodder) in many districts, which contributed to their high prices (% price hike ranging between 9 to 16%). Impact on labor supply for agricultural activities was uneven across the state: reduced labor supply was reported in 10 districts, while increased availability was reported in 8 districts. This was attributed to the return of migrant labor in their native villages. The demand for farm labor, however, reduced in many districts during the pandemic.

##### Concerns Regarding Future Crop Sales and Farming Patterns

The findings from telephonic survey revealed varying challenges in the three communities regarding their perception of future impact of COVID-19 pandemic on their farming patterns. During the lockdown phase, more than half the HHs in Munda and Santhal communities were concerned that the continued closure of markets might affect their future ability to purchase seeds, fertilizers, and other equipment necessary for crop production (Table 2). However, during the unlock phase, relatively fewer HHs in both communities were concerned regarding the purchase of external inputs for farming. Further, during the lockdown phase, HHs in all Santhal and Munda communities were concerned about the future availability of manual labor for assistance in farming due to the fear of COVID-19 virus spread and the consequent higher labor charges. HHs in all three communities reported concerns about their future crop sales, owing to the continued closure of local markets and movement restrictions across districts and states, which they perceived as likely to impact their ability to sell their farm and forest produce. A male respondent from Sauria Paharia community commented, “As *the markets are closed, fewer people from the villages will buy the (farm)produce, so it will be difficult to sell”* while another male respondent from the Santhal community opined *“Because of reduced access to labor and seeds, our crop yield might get affected, which may lead to reduced crop sales.”* This concern over future selling ability continued in unlock phase as well, with a higher number of HHs (particularly in Sauria Paharia and Munda communities) anticipating a reduction in their future crop sales, which was attributed to an increase in the number of market vendors selling their farm produce (Table 2).

### Impact on Food Access

#### Direct Impact on Food Access Due to COVID-19 Pandemic and the Resulting Lockdown

##### Impact on Informal Weekly Markets

**Figure 6:**
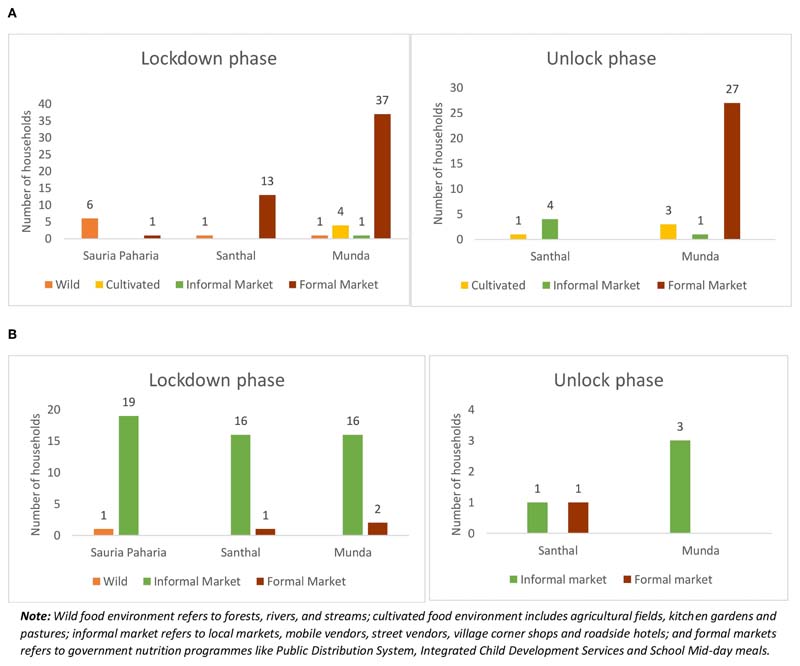
Impact of COVID-19 pandemic on household food access in indigenous communities of Jharkhand, India. **(A)** Number of HHs citing places that were easier to access for food during the lockdown phase (May-June, 2020) and the unlock phase (September-October, 2020). **(B)** Number of HHs citing places that were difficult to access for food during the lockdown phase (May-June, 2020) and the unlock phase (September-October, 2020).

**Figure 7:**
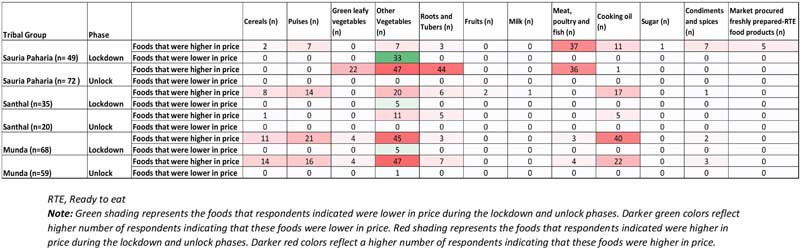
Impact of COVID-19 pandemic on food prices in tribal regions of Jharkhand, India.

**Figure 8:**
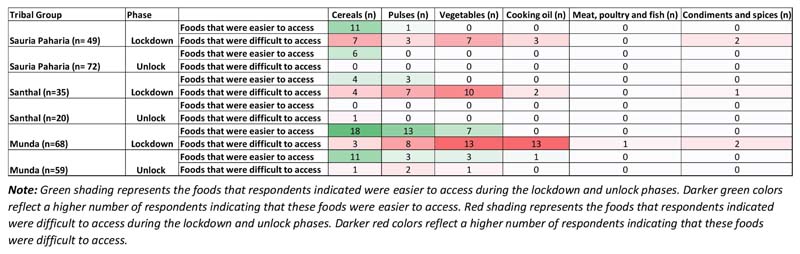
Impact of COVID-19 pandemic on food access in different indigenous communities of Jharkhand, India.

During the lockdown phase, a substantial number of HHs (63%) in all three indigenous communities observed changes in their access to different food environments and sources, with the highest impacts reported by the Munda community, followed by Santhals and Sauria Paharias (Table 2). Among these communities, about one-third of the HHs (highest in the Santhals) reported hardships in procuring food items from the local informal markets (Figure 6). Common reasons cited for reduced access included reduced opening hours (early mornings or late evenings) of the markets amidst the lockdown, limited diversity in available food commodities, increased food prices, and lack of transportation facilities to reach the local markets. When enquired about the impact of COVID-19 pandemic on the food prices, most HHs (87%) in all three communities observed differences in the usual pricing of the food items (Table 2). The respondents attributed this change to the sudden announcement of the lockdown, which led to restricted movement and hampered the timely reach of food supplies to the local market, thus leading to price fluctuations. More than half of the HHs in Santhal and Munda communities reported an increase in the prices of vegetables. In contrast, the majority of Sauria Paharia HHs reported a decrease in the prices of vegetables, owing to an improved availability through the cultivated food environment and distress selling of these commodities by the local vendors. Apart from vegetables, other food commodities like meat, poultry and fish, pulses, and cooking oils were also reportedly being sold at inflated prices, as compared to the pre-COVID-19 period (Figure 7). As a consequence, HHs, during the lockdown phase, reported decreased access to specific food items, particularly specific vegetables (20%), pulses (12%), and cooking oil (12%), from the nearby local markets. On the other hand, improved access to cereals (mainly rice) was reported in some HHs of Munda (27%) and Sauria Paharia (22%) communities (Figure 8).

During the unlock phase, despite easing of lockdown restrictions and resumption of the flow of food through supply networks, a large proportion of HHs (90%) in all three communities reported an increase in prices of food items, which mainly comprised of perishable foods like green leafy vegetables, meat, poultry and fish and other vegetables (Figure 7). However, despite the inflation in food prices, the food access during this period was reportedly similar to pre-COVID-19 period in majority of the HHs (Figure 8).

##### Impact on PDS and Other Government Food Security Programs

During the lockdown phase, improved access to formal markets (especially the PDS) was reported in one-third of the total HHs, especially among Santhal and Munda communities (Figure 6). Nearly, 50% of the HHs reported receiving additional amounts of food grains (rice and pulses) through PDS during the lockdown. As one male respondent from Sauria Paharia community commented *“Rice and pulses are now more easily available as government is providing ration at home and for free.”* Among Santhal HHs about 15% also reported access to hot-cooked meals for the entire family *via* the school feeding program (MDM). An additional one-fourth of the HHs in Santhal and Munda communities reported availing take-home ration from ICDS. During the unlock phase, the overall access to the formal markets was better for PDS, with 95% of surveyed HHs in all three communities reporting accessing the fair price shops and also receiving additional rations. However, fewer HHs from all three communities reported receiving additional amounts and/or varieties of food grains from PDS during the unlock phase (25%) as compared to the lockdown period (50%) (Figure 6). Additionally, a relatively lower proportion of surveyed HHs from Santhal (10%) and Munda (6.8%) communities reported receiving take home ration from ICDS (Figure 6).

**Figure 9:**
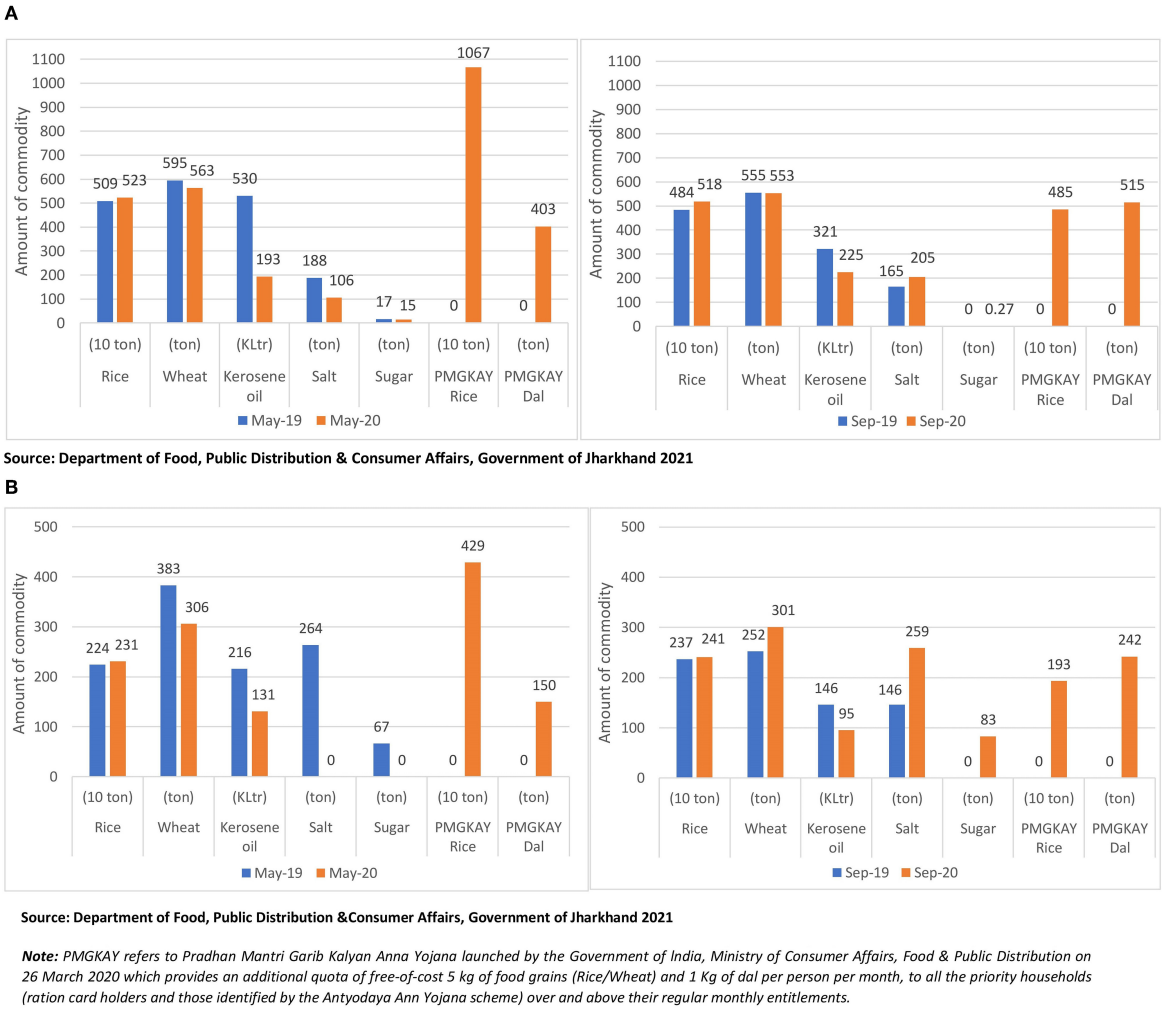
Distribution status of communities under PDS in study districts of Jharkhand. **(A)** Transaction status of various commodities under PDS during pre-covid time (May 2019) v/s lockdown time (May 2020) and pre-covid time (September 2019) v/s unlock time (September 2020) in Godda, Jharkhand. **(B)** Transaction status of various commodities under PDS during pre-covid time (May 2019) v/s lockdown time (May 2020) and pre-covid time (September 2019) v/s unlock time (September 2020) in Khunti, Jharkhand. Source: Department of Food, Public Distribution and Consumer Affairs, Government of Jharkhand, 2020.

The secondary data on access to government food security programs in selected study districts of Jharkhand, revealed similar outcomes during the lockdown and unlock phases. Higher amounts of rice were distributed through PDS during both lockdown and unlock phases in both Godda and Khunti districts, with additional amounts of rice and pulses distributed by Pradhan Mantri Garib Kalyan Anna Yojana (Figure 9). However, in the case of other commodities like wheat, kerosene oil, salt, and sugar, comparatively lower amounts were distributed during the lockdown phase in both districts, while higher amounts of wheat, salt, and sugar were distributed during the unlock phase in Khunti district. According to the State audit data for Godda district (State Food Commission Social Audit Unit Jharkhand, 2020), (conducted between 6th-17th May, 2020), utilization of MDM and ICDS programs was reportedly better than the data collected from indigenous communities of Godda district in the present study during the lockdown phase.

According to the secondary data, only about 29% HHs did not receive food grains via MDM and 46% HHs did not receive food grains through ICDS. Secondary data on the audit of MDM and ICDS utilization was not available for the Khunti district.

#### Indirect Impact of COVID-19 Pandemic and Resulting Lockdown

Apart from directly affecting the food supply chains during the lockdown, survey findings indicate that the COVID-19 pandemic also influenced people's access to food through loss of livelihoods and income (Table 2). During the lockdown phase, more than three-fourths of the surveyed HHs reported a decrease in HH income, among which about 25% of the HHs lost their livelihood source altogether. Surveyed HHs from the PVTGs observed the highest impact on income i.e., the Sauria Paharias (86%), followed by the Santhal (80%) and the Munda (69%) communities (Table 2). Reduction in crop sales was the main concern among the communities, with significant changes experienced by the Sauria Paharias (67%). Closed markets, movement restrictions, and lack of transport facilities during the lockdown were cited as the common reasons for reduced crop sales in all three communities. One male respondent from Santhal community stated *“I cannot sell crops now as the local markets are closed and I have no other source of income*,” while another male respondent from Munda community was worried about not being able to travel for work. He shared *“I cannot sell my farm produce due to the closed markets and neither can I migrate for work because of travel restrictions.”* A female respondent from Sauria Paharia community mirrored similar views and stated "We *have no work now, so there is no money. We can't earn money from farming (sale of crops) also. Everything is closed due to coronavirus pandemic. Earlier we used to sell Barbatti (cowpea) and use the money to buy food, but due to lockdown, we are unable to sell, hence there is no earning.”* Consequently, more than half the HHs (57%) among Sauria Paharias and one-fifth HHs in Santhal and Munda communities resorted to selling their crops at lower prices (as compared to previous years) as limited people were accessing the local weekly markets. Further, to improve their HH income, a large majority of the HHs from the Sauria Paharia community (76%) reported selling wild products (sourced from surrounding forests and wild habitats) in the weekly markets. One of the male respondents shared: *“Due to coronavirus pandemic (lockdown), we have started sellingfruits-Mahua (Indian butter tree) and Aam (Mango) in the local markets. We have also started collecting leaves from the forests, which are woven and sold as leaf plates. We use the money earned from selling these items to buy food for household consumption.”* A small proportion of HHs (14%, *n =* 5/35) from the Santhal community also reported this practice. The Munda community did not report this practice.

During the unlock phase, some HHs reported improvements in financial conditions as compared to the lockdown phase. However, about 40% of HHs reported a reduction in HH income, out of which around 6% HHs completely lost their livelihood source. This impact was faced uniformly across the three communities (Table 2). As the mobility restrictions were eased during the unlock period, half the HHs (50%, *n* = 36/72) in the Sauria Paharia community, and a few HHs in Santhal (*n* = 4/20) and Munda (*n* = 6/59) communities reported that they resumed selling their farm produce at usual market prices or even higher than usual prices (as compared to pre-COVID-19 period). However, the practice of accessing the wild environment to sustain HH income declined during this phase. During the countrywide unlock phase, as the employment opportunities improved, only a couple of HHs (two HHs in Sauria Paharia and one HH in Munda community) reported selling the forest produce in the local markets.

#### Community Perceptions on Their Future Food Security Status

During the lockdown phase, the majority (59%) of the HHs from the three indigenous communities expressed concerns regarding different aspects of food security in the future (Table 2). Sauria Paharia, the most vulnerable community among the three communities surveyed, did not report any concern regarding food availability; the main concerns among a majority of the HHs (51%) in this community were related to food affordability owing to the increased food prices. HHs in Santhal and Munda communities reported concerns related to both food availability (44%) and affordability (45%), while some HHs also stated concerns regarding safety (18%) and quality (6%) of food during the pandemic, as they were worried about contracting COVID-19 infections through the foods available in the local markets (Table 2). During the unlock phase, relatively fewer HHs (35%) were worried about aspects related to food security. However, in the Munda community, the majority of HHs (75%) were worried about their future HH food security status, with major concerns around the future availability and affordability of foods. Among Santhals, a limited number of HHs expressed concerns on the future status of HH food availability, while none of the HHs in the Sauria Paharia community reported any concern on either of the food security aspects.

### Impact on Utilization of Food

**Figure 10:**
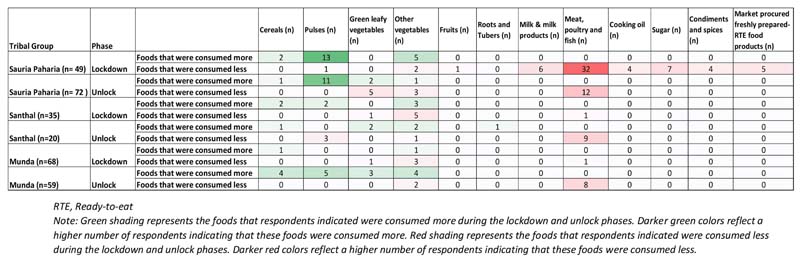
Impact of COVID-19 pandemic on food consumption pattern in tribal regions of Jharkhand, India.

In all three indigenous communities, the changes in food access (albeit, in relatively lower magnitude) were further reflected in the consumption patterns of the HHs. During the lockdown phase, almost half (43.4%) of the HHs reported a change in food consumption patterns, with major changes experienced by the HHs in Sauria Paharia (76%) and Santhal (49%) communities. A substantial number of HHs in Sauria Paharia and Santhal communities reported reduced consumption of meat, poultry and fish and market procured freshly prepared sweets and savories (Figure 10). According to the respondents, before the onset of the COVID-19 pandemic, meat, poultry and fish were consumed once every week which was restricted to only once a month during lockdown restrictions. The change was mainly attributed to high food prices and diminished food affordability of the HHs. A couple of Sauria Paharia HHs (*n* = 2/49) also perceived that meat, fish, and poultry consumption may lead to COVID-19 infection. The Santhal HHs further reported consuming a less diverse diet consisting of mainly plain rice to save and stock up the other food items for future consumption. One Santhal respondent commented, *“Before we used to eat rice, pulses, and vegetables, but now we are consuming only plain rice with rice water,”* while another respondent shared, *“We cannot eat pulses now. We only eat plain rice with mango chutney (paste) nowadays.”* Although no major variations were observed among Munda HHs, some HHs (*n* = 5/59) reported consuming all food items in reduced quantities, due to their poor purchasing capacity during the lockdown.

The surveyed HHs in all three indigenous communities also reported a higher intake of certain food items during the lockdown phase. A small proportion of HHs (in Sauria Paharia and Santhal communities) reported stocking up on the fresh farm and kitchen garden produce, to meet their overall food requirements. These activities were undertaken to mitigate the effect of reduced HH earnings and food price inflation in the local markets. For instance, more than one-third of the HHs in Sauria Paharia community reported higher consumption of farm-produced grains (rice and maize), home-grown indigenous pulses (like Horse gram, Cowpea, Rice bean, and Red gram), and wild leafy vegetables collected from the forests. During the unlock phase, only a fifth of the total HHs reported changed consumption patterns. A relatively lower number of Sauria Paharia and Munda HHs reported reduced consumption of meat, poultry and fish and a couple of Munda HHs (*n* = 6/59) demonstrated reliance on their farm and kitchen garden produce to meet their day-to-day consumption needs (Figure 10).

### Impact of COVID-19 on Informal Markets of Jharkhand, India

#### Impact on Food Prices and Retail

**Figure 11:**
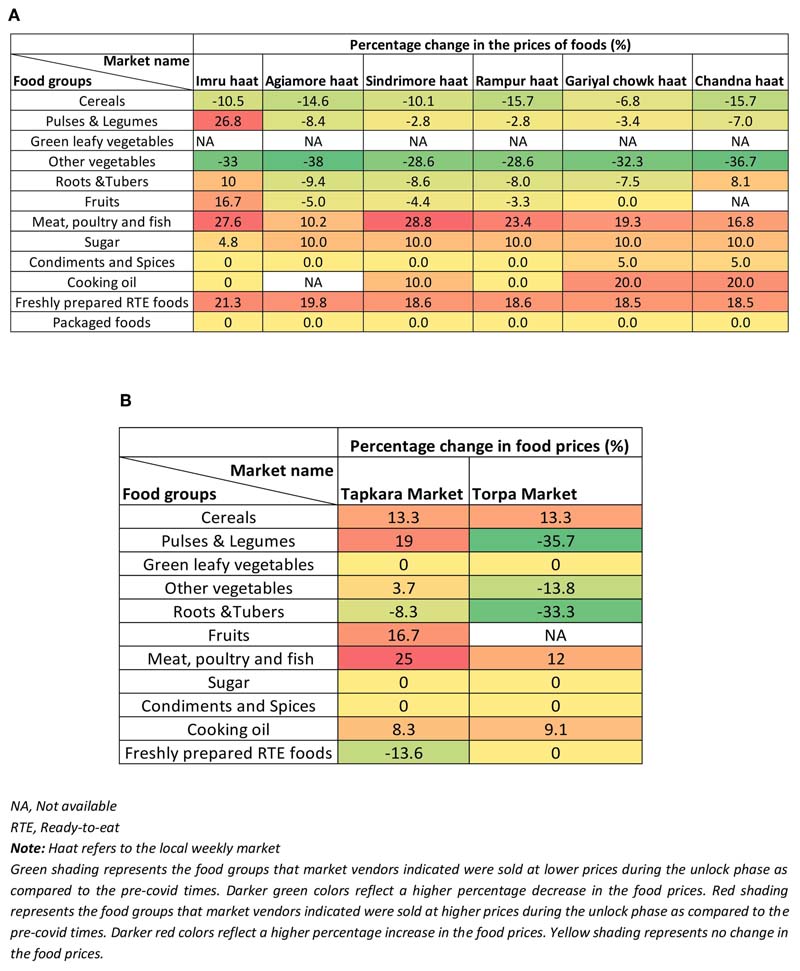
Impact of COVID-19 pandemic on the retail prices of the food groups in the informal markets in indigenous communities of Jharkhand, India. **(A)** Impact of COVID-19 pandemic on retail prices of food groups in the informal markets of Godda district, Jharkhand, India, catering to Santhal and Sauria Paharia communities. **(B)** Impact of COVID-19 pandemic on retail prices of food groups in the informal markets of Khunti district, Jharkhand, India, catering to Munda community.

Based on the market surveys conducted (in August 2020) during the third unlock phase in India, notable impacts of the COVID-19 pandemic, and mitigation measures were observed on the prices and sales of food items available in the informal markets of Godda and Khunti districts in Jharkhand. In the local informal markets of Godda district (*n* = 6) that cater to Sauria Paharia and Santhal communities, decreased prices of indigenous cereals (rice, maize, and pearl millet) were reported (Figure 11A), due to better food distribution through PDS during the pandemic, resulting in lower consumer demand for the food grains. As a result, vendors in 2 out of the 6 surveyed markets reported reduced sales of cereals. Some varieties of indigenous pulses (including horse gram and cowpea) and vegetables (including kovai and bittergourd) were reportedly being sold at lower prices in all the six markets surveyed, due to their better availability via the local cultivated food environment. Owing to continued restrictions on wage laboring and allied jobs during the unlock phase, many HHs had resorted to distress selling, which, in turn, contributed to surplus availability of indigenous vegetables and pulses. However, despite the reduction in their prices, reduced sales of pulses and vegetables were reported (in 4 out of 6 markets), owing to market restrictions that continued even during the unlock phase of 2020. Concurrently, a retail price hike was reported for market-based pulses (including lentils, red gram, green gram, and chana dal) in Imru market, and for meat, poultry, and fish in all the markets due to an impact on the food supply chains during the pandemic (Figure 11A), which led to reduced sale of these foods. Among the roots and tubers food category, potatoes were being sold at higher prices than usual in 3 out of 6 markets, however, onions were being sold at lower prices in 5 out of 6 markets, while prices of ginger and garlic remained the same. The sale of very limited fruit varieties was reported in all the six markets, with a slight reduction in their prices. An increase in the prices of cooking oil (3 out of 6 markets), sugar (all the 6 markets), and a slight increase in the prices of condiments and spices (2 out of 6 markets) were observed. The prices of some of the freshly prepared ready-to-eat sweets and savories (*jalebi, rasgulla, aloo chop)* increased in the range of 18.5 to 21.3% (Figure 11A), however, no changes were observed in their sales.

In the local markets of Khunti district (*n* = 2) (catering to Munda community), only hybrid rice varieties were available, which were being sold at higher prices (percentage increase by 13.4%) (Figure 11B) due to the disrupted supply chains. Similar to informal markets of Godda district, the market vendors in Khunti district reported a price hike for market-based pulses (in Tapkara market) and reduced prices of indigenous pulses (in Torpa market) and other vegetables, In the case of roots and tubers, variable changes were observed in the prices of potato and colocasia across the two markets; however, garlic and ginger became cheaper. Price inflation was observed for meat, poultry and fish across all markets (percentage increase of 12% to 25%) (Figure 11B) due to supply chain disruptions and reduced consumer demands. Prices of packaged foods and freshly prepared foods remained the same (except for *samosa),* yet their sales were significantly reduced, due to the low consumer demand during the pandemic.

#### Perceived Impacts on Market Vendors and Their Earnings

We further observed impacts on the income and livelihoods of the local vendors in the local markets of the Godda and Khunti districts of Jharkhand. Due to fear of the COVID-19 virus, shutting down of markets, and reduced sales, a relatively small number of vendors were selling cereals, vegetables, and meat, poultry and fish in the markets catering to the Sauria Paharia and Santhal communities (*n* = 6). In almost all of the surveyed markets (5 out of 6 markets), fewer vendors were selling freshly prepared sweets and savories than usual as they couldn't afford the raw materials for preparation (owing to a hike in their prices). In markets catering to Munda community (*n* = 2), a large number of people were selling vegetables due to their surplus stocks from local produce, while on the other hand, a smaller number of vendors were selling meat, poultry and fish, packaged foods, and freshly prepared savories and snacks owing to their higher retail prices and reduced demand.

When inquired about the impact of COVID-19 pandemic on their income, the Godda and Khunti districts' market vendors reported similar changes. The vendors selling cereals, pulses, and vegetables in both districts reported reduced incomes, and cited factors like improved access to PDS and closed markets as the main reasons for income loss. On the other hand, vendors selling meat, poultry and fish, packaged foods, and freshly prepared foods in markets of Godda district reported an increase in their overall income owing to increased prices of the food items, in comparison to the pre-COVID-19 period. However, in Khunti district, these vendors experienced relatively lower incomes (in comparison to the pre-COVID-19 period) owing to the reduced consumer demand for these foods.

### Factors Offering Resilience During the COVID-19 Pandemic

Based on the responses from the HHs, certain practices of the indigenous communities were found to demonstrate resilience in response to the impact of COVID-19 pandemic on their food consumption patterns, income generation, and farming systems. Among Sauria Paharias HHs, access to the wild and cultivated food environment was the primary factor that offered resilience in the face of the COVID-19 pandemic. Through our C-SCAN survey findings, it was observed that several Sauria Paharia HHs relied on wild edible plants from their local wild habitats as well as produce from the *Kurwa* lands and kitchen gardens for fulfilling their day-to-day food consumption needs. They also utilized the wild food environment for additional income generation by selling the wild produce in the local markets. Another notable factor bolstering the resilience of HH in response to COVID-19 pandemic in our study was the improved supply chain for formal food markets, especially the government-supported program of PDS.

The transport disruption and market closures associated with the COVID-19 pandemic led to hardships among Santhal and Munda HHs in procuring farm inputs. However, the Sauria Paharia HHs, a large proportion of whom still relied on indigenous seeds (Ghosh-Jerath et al., 2020, 2021), were relatively less impacted during the lockdown and post lockdown phase compared to the other surveyed communities. The community utilized local inputs i.e., their indigenous seeds and compost, during the sowing season. Further, HHs in the community reported better access to labor during the sowing season, owing to the back migration of individuals returning to the community from their place of work in urban areas. One respondent stated, *“Earlier, we used to hire manual labor but since everyone is at home due to the lockdown, the entire family is working on the farms because we don't have the money to hire labor during this period.”* In addition to this, a better access and dependence on diverse food sources (i.e., access to wild and cultivated food environment) among all three indigenous communities, offered them resilience and facilitated better food security with reference to food production, availability, and access during the lockdown phase of the pandemic. These findings concurred with the factors highlighted in the conceptual framework that may offer resilience in the context of shocks to the food system (Figure 3).

## Discussion

Administration of a rapid tool (C-SCAN) based on a food environment typology framework along with a market survey in Jharkhand state of eastern India highlighted how the COVID-19 pandemic adversely impacted the ability of the indigenous communities to procure sufficient, affordable, and nutritious foods while highlighting resilience attributes of indigenous food systems for supporting food security. Findings from the market surveys revealed variability in prices of commodities: reduced supplies led to a price hike while lower consumer demand resulted in reduced prices. Better outreach of PDS, access to diverse food environments and locally produced food resources were identified as critical factors for enhancing the food security of the surveyed communities in the context of the COVID-19 pandemic. However, some of the supplementary feeding programs were sub-optimally utilized due to the closure of the distribution platforms.

### Impact on Agricultural Practices, Food Production and Supply Chains

During both the lockdown and unlock phases of the COVID-19 pandemic control measures, the majority of the study respondents did not report any effects on agricultural yields. Similar findings were observed in our secondary review, which highlighted minimal impacts on farm production in Jharkhand, although the allied sectors (e.g., horticulture) were adversely affected. The Munda and Santhal communities reported facing challenges in the procurement of farm input supplies during the lockdown phase, while a few respondents from the Sauria Paharia community reported the practice of early sowing due to availability of family members, owing to reverse migration. Similar disruptions in food production and availability of farm inputs due to transport restrictions have been reported in other studies across India (Harris et al., 2020; Nandi et al., 2021). The concerns regarding lack of manual labor during harvesting and sale of produce (especially perishable commodities) have also been reflected in data from the Indian states of Bihar, Rajasthan and Maharashtra (Kapil, 2020). This could be due to the absence of public procurement institutions for agricultural inputs as well as the labor-intensive cultivation among the smallholder farmers. The experiences of the indigenous smallholder farmer communities in India could hence be useful for designing future relief packages, responsive to the cropping patterns, local availability of farm inputs, labor and access to markets. This could be a crucial step toward building of resilient farming systems in the face of similar future shocks (Ceballos et al., 2020).

### Impact of Supply Chain Disruptions on Built Food Environment

A distinct variability was reported by all the three communities as well as the market vendors regarding the availability of food commodities from the built food environment, especially the informal markets with price inflation for specific food commodities (including pulses, meat, poultry and fish, oils, some vegetables) due to supply chain disruptions on one hand, and higher availability of local perishable produce (especially vegetables) leading to distress selling, lower demand and resulting lower prices. Disrupted access to informal markets has been reported in other studies, as is supply chain disruption leading to a decrease in the availability of vegetables and fruits in these informal markets (Harris et al., 2020; Mahajan and Tomar, 2020). Further, adverse impacts on weekly informal markets were reported in almost all surveyed districts in Jharkhand, during the lockdown phase (NABARD, 2020). Studies have documented both distress selling for income generation (The New Indian Express, 2020; Cariappa et al., 2021; Rao, 2021), and price hikes in food commodities ascribed to movement restrictions that also limited arbitrage possibilities across cities after the lockdown (Narayanan and Saha, 2020; Sukhwani et al., 2020; Varshaney et al., 2020). The variable changes in commodity prices during the lockdown phase were likely driven by the nature of the commodity e.g., vegetables/fruits in terms of their availability from nearby regions or being a market-based commodity (like non-indigenous pulses and meat, poultry and fish) dependent on transportation networks and *mandi* inventories. Shorter supply chains can not only provide a buffer during such global crises, due to their rooted presence in the region, and proximity to the consumers but may provide a boost to the local micro-economy as well (Cappelli and Cini, 2020). This is particularly pertinent in the Indian scenario where there is a huge dependence on the agrarian economy (50% of India's total workforce) making the localized food chain, food processing, and allied sectors the bulwark for an uninterrupted food supply (Thulasiraman et al., 2021).

### Improved Access to Formal Markets and Government Food Security Programs

In contrast to variations in access to food in informal markets, improved access to formal markets was reported for government-supported programs like the PDS (Kaur, 2020), and in some cases from the school meal program i.e., MDM. This improved access was reflected in almost all HHs accessing the fair price shops and other COVID-19 welfare schemes like Pradhan Mantri Garib Kalyan Yojana. Similar trends were reported for access to formal markets under government programs especially the PDS in many other states, reflecting an improved outreach (Lahoti et al., 2020; Pande et al., 2020) and was of immense importance in providing relief during the various lockdown phases (Sinha, 2021), despite the challenge of reaching all segments of the population consistently. While the additional provision of cereals and pulses through PDS was important for ensuring food security, it was not a sufficient measure to address the widespread prevalence of micronutrient malnutrition among the indigenous communities, that may have worsened during the pandemic (Gupta et al., 2021). Further, provision of non-perishables like rice, wheat and pulses, instead of diverse nutritious foods, may have further reinforced the consumption of cereal-based monotonous diets lacking in essential nutrients (Headey and Ruel, 2020). It is thus important to include nutrient-dense foods like millets (finger millet, sorghum) in the distribution basket of PDS, which may prove beneficial in improving the diet quality and nutritional status of these vulnerable populations, especially in situations when market access and income flow is severely affected (Gupta et al., 2021).

Though our study communities reported reduced access to supplementary feeding programs like ICDS, the district level data indicated a better utilization. This discrepancy could be attributed to the poor access to hard-to-reach villages, thus resulting in poor uptake ofthe specific schemes and interventions during the pandemic owing to center closures, supply chain disrruptions, and in some cases, repurposing of the local frontline workers for COVID-19 pandemic mitigation efforts like awareness generation, mask distribution and production, etc (KMPG, 2020). The disruption in the ICDS and MDM program as reported in our study and other reports may have a long-term impact on the nutritional status of vulnerable children who depend on these feeding programs to meet their nutritional needs (Bahl et al., 2021). Hence, it is important to keep these programs operational and continue the distribution of additional food and micronutrient supplements for pregnant women, adolescents and children, by strengthening the delivery systems while adhering to the social distancing norms (Haque et al., 2020).

### Disruption in Livelihoods

The lockdown and the staggered unlock phases resulted in significant impacts on the livelihoods and incomes of the majority of respondents, with Sauria Paharias reporting the largest impact. These losses were attributed to a reduction in the sale of agricultural produce, or distress selling at lower prices. Similar findings were highlighted in a nationwide survey on informal workers that reported a high level of unemployment in Jharkhand during lockdown (95%) and unlock phases (62%), which resulted in indebtedness in nearly one-fourth of the population during both phases (Action Aid, 2020). Other studies have documented a loss of daily wage jobs in the range of 45−65% among rural migrants from Bihar and Jharkhand (Imbert et al., 2020; Save the Children, 2020), during the lockdown and post-lockdown period. The role of MGNREGA (Mahatma Gandhi National Rural Employment Guarantee Act) was found to be limited in this context; only 30% of the registered ST HHs in Godda and Khunti districts of Jharkhand, received employment during the entire period of 2020 (Mahatma Gandhi National Rural Employment Guarantee Act Jharkhand, 2020). In our study, most HHs in Santhal and Munda communities who suffered a loss of income during the lockdown phase were able to return to their usual ways of livelihood, but a considerable share of Sauria Paharia HHs continued facing difficulties in improving their income flow during the unlock phase. Studies suggest that prolonged income losses in such vulnerable communities may further lead to negative coping strategies, like distress sale of assets, predatory loans or child labor (WHO, 2020; ILO and FAO, 2021). It is thus essential to safeguard the vulnerable populations with stable cash flow through strategies like direct cash transfer programs and strengthening of existing employment guarantee schemes (like MGNREGA) (Dev, 2020).

### Impact on Dietary Intake

A significant proportion of the respondents from all communities in our study reported a change in their dietary intake patterns with a decrease in dietary diversity. An increase in consumption of certain foods, especially locally grown indigenous produce, was also reported. Similar to our study findings, other studies from India and elsewhere have reported an impact on overall diets and a decrease in diet diversity scores of the communities (Harris et al., 2020; Bauza et al., 2021; Kansiime et al., 2021; Kusuma et al., 2021). Some reports suggest similar patterns of increased consumption of vegetables but a decrease in consumption of meats and dairy; others report dietary changes linked to food security status (Cicero et al., 2021; Litton and Beavers, 2021). It is worth highlighting here that the ability to consume one's own produce can be somewhat protective toward ensuring dietary diversity when other food access options are compromised.

### Factors That May Offer Resilience to Food Systems Shocks Such as the COVID-19 Pandemic

Our telephonic survey provided a snapshot of various dimensions of food security that were affected in the study communities. While the COVID-19 pandemic situation posed many challenges both during and after the lockdown, we have identified several attributes in the survey responses that could offer resilience and inform preparedness for such unprecedented calamities in the future. One of the attributes that decreased the negative impact of the COVID-19 pandemic and associated mitigation measures on agricultural productivity among the Sauria Paharias was the availability of family members for sowing and farming practices. The increased use of family labor may incidentally provide some relief to smallholder farmers to overcome possible labor shortages around harvesting, getting food to market, and other farm-related activities (Haga, 2020). Recently, the Food and Agriculture Organization (FAO) has recognized family farmers as the custodians of multi-cropping systems, who may have the potential to enhance HH nutritional security, improve resilience to crop failures and price shocks, reduce migration and eradicate poverty. However, to turn this potential into reality, family farming needs an enabling policy environment, that promotes their access to natural resources and provides them with employment and social protection. With FAO's involvement in different family farming projects across the globe, this opportunity could be leveraged to raise awareness, scale-up support and enhance the capacities of local institutions and organizations for implementation of integrated family farming and rural developmental strategies (FAO, UN, 2018).

The Sauria Paharia's traditional practice of sowing indigenous varieties of seeds also offered resilience in the context of seed shortages since they were not exclusively dependent on the market availability of these farm inputs. The improved access to government-supported programs like PDS through formal food markets also proved to be a critical support measure. Our findings highlight that the traditional attributes of food systems need to be strengthened further and activities such as providing THR at the doorsteps of HHs during catastrophes need to be enhanced. In addition, the mitigating factors that addressed lower dietary intakes identified in the present study could serve as leverage points for the future. For certain food items in the local markets e.g., vegetables, the shorter supply chains were demonstrated to be beneficial in maintaining their availability. In fact, there were issues around the decrease in the availability of specific food items like oils and meat, poultry and fish as they were either cultivated or processed farther from the final point of sale (Mahajan and Tomar, 2020). It is important to recognize that while indigenous smallholder farmers are vulnerable with respect to their geographical location, they can also demonstrate resilience with respect to their food systems. Thus, these smallholder farmers can potentially be the most important providers of food in contexts where the need to enhance food security is greatest. Further, they can also effectively serve domestic markets, especially at times when trade is compromised. Smallholder farmers are wellplaced to continue the supply of food in situations where the COVID-19 crisis has created complex logistical and transport issues (Haga, 2020). An increase in foraging of wild foods also supplemented the diets of our study communities and provided an additional source of income. Although studies from Africa and Latin America (Blaney et al., 2009; Powell et al., 2011; Termote et al., 2012) have documented the role of agroforestry systems in enhancing the dietary diversity, evidence on contribution of wild foods and tree-based agriculture systems toward nutrition and livelihood security among smallholder farm communities, remains largely under-researched. In our study findings, we observed a clear dependence of the communities on the natural environment for food and livelihood at the time of crisis. Hence, a greater focus on sustainable use of wild produce will be crucial in initiating global efforts toward a more food secure and nutritionally sensitive future among vulnerable populations (Sunderland et al., 2013).

### Study Limitations

Since the present study was based on a telephonic mode of data collection, there were a few limitations that may have impacted our study findings. First, as our study sample resided in hard-to-reach areas, most of the HHs could not be contacted due to poor network and call failures. Nonetheless, the possibility of a high non-response rate was considered while calculating the study sample size. Second, the telephonic nature of the survey often resulted in respondent fatigue, which may have influenced their responses. Third, although the study team had field staff speaking the local language, language and cultural barriers may have influenced some responses. Fourth, in the case of HHs where women were principally involved in food production and/or collection and preparation, telephonic interviews with male respondents may have influenced our study findings to some extent. Fifth, the purposive selection of study samples for both telephonic and market surveys may have induced some researcher bias, that could have an impact on the generalization of our study findings. However, we have tried to address this limitation by triangulating our study findings with secondary data from state and district level reports. Lastly, as the present study was a serial cross-sectional study, our findings cannot be used for establishing causality, drawing inferences at the individual level and effects of HH-level attributes toward the study results and resilience. However, the findings have provided some crucial information on how the COVID-19 pandemic has variably affected the food systems of the three indigenous communities. The mixed-methods approach has further been able to capture the community-level factors that may offer resilience in the context of food availability, access, and utilization.

## Conclusion

This study highlights how the COVID-19 pandemic affected the ability of indigenous communities in Jharkhand state of India to procure sufficient, affordable, and nutritious foods while aspects of the indigenous food systems of the surveyed communities displayed crucial features of resilience to support the key pillars of food security, especially access and stability. Key drivers of the adverse impacts of food access for the surveyed communities were restrictions in the movement of farm labor and supplies, along with disruptions in food supply chains and other food-related logistics and services associated with the COVID-19 pandemic and mitigation measures. Key determinants of resilience for the surveyed communities were the ability to access diverse food environments, particularly wild and cultivated food environments, indigenous farm inputs and the improved access to fair price shops when local informal markets experienced shocks to food supply and shifts in prices. Findings highlight the critical need to support biocultural diversity in indigenous communities. These would include conservation of biodiversity of forests and other wild habitats as well as indigenous knowledge systems and associated practices and resources such as indigenous seed cultivars to propagate and sustain a rich diversified sustainable ecosystem of foods. Strengthening of food security and employment welfare schemes is further imperative to minimize the impacts on food and livelihood insecurity in vulnerable populations. Building food systems that are resilient to shocks such as the COVID-19 pandemic, requires shorter agri-food supply chains dependent on local and regional food sources, with collective action among all the stakeholders, including the agricultural extension services, food retailers, policymakers, governments, as well as the consumers. Findings on how indigenous communities tapped into their traditional foods systems in the context of the COVID-19 pandemic provide an opportunity to better understand the consequences of a global pandemic on the food and livelihood security of the vulnerable populations. Programs and interventions are called for to conserve and revitalize the biocultural resources available within these vulnerable communities thereby supporting healthy, equitable, and sustainable food systems for all. We should aspire to grow back more harmoniously with our environment as we endeavor to build back our world after the pandemic.

## Supplementary Material

Table S1

Table S2

## Figures and Tables

**Table 1:** General profile of telephonically surveyed households belonging to indigenous communities of Jharkhand, India.

Characteristics	Lockdown phase (May-June, 2020) (*N* = 152)	Unlock phase (Sept-Oct, 2020) (*N* = 151)	*p* value^†^
**Age of the respondent [Mean (SD)]**	33.2 (±11.2)	31.52 (±8.8)	0.148
**Gender of the respondent, *n* (%)**			0.764
Male	116 (76.3)	113 (74.8)	
Female	36 (23.7)	38 (25.2)	
**Households, *n* (%)**			0.011**
Sauria Paharia	49 (32.2)	72 (47.7)	
Santhal	35 (23.0)	20 (13.3)	
Munda	68 (44.8)	59 (39.0)	
Type of farming*, *n* (%)			0.000**
Only Farm	18 (11.9)	30 (19.9)	
Only Kurwa	24 (15.8)	47 (31.2)	
Only Bari	3 (2.0)	1 (0.7)	
Any two sources	97 (63.9)	71 (47.0)	
All three sources	95 (62.5)	–	
None	3 (2.0)	2 (1.4)	
**Primary source of income, *n* (%)**			0.939
Agriculture	110 (72.4)	112 (74.2)	
Daily wage labor	28 (18.4)	26 (17.2)	
Others (own business, remittances, etc)	14 (9.2)	13 (8.6)	

* Multiple responses were captured as households practiced multiple types of farming.

** *p* < 0.05.

† Continuous variable (age of the respondent) following a normal distribution between the indigenous communities were compared using independent *t*-test and the remaining categorical variables were compared using Chi-square test.

**Table 2:** Perceived impacts of COVID-19 pandemic on food production and access among indigenous communities of Jharkhand, India.

Components	Lockdown phase (May-June, 2020), *n* (%)				Unlock phase (Sept-Oct, 2020), *n* (%)				*p* value^†^
	All indigenous communities (N = 152)	Sauria Paharia (N = 49)	Santhal (N = 35)	Munda (N = 68)	All indigenous communities (N = 151)	Sauria Paharia (N = 72)	Santhal (N = 20)	Munda (N = 59)	
**Change in overall food production**	12 (7.9)	3 (6.1)	5 (14.3)	4 (5.9)	4 (2.7)	–	1 (5)	3 (5.1)	0.394
**Type of change**									
1. Negative impact^$^	11 (7.2)	3 (6.1)	5 (14.3)	3 (4.4)	2 (1.4)	–	–	2 (3.4)	
2. Positive impact^$^	1 (0.7)	–	–	1 (1.5)	2 (1.4)	–	1 (5)	1 (1.7)	
**Changes in sale of farm produce**	66 (43.5)	33 (67.4)	12 (34.3)	21 (30.9)	60 (39.8)	39 (54.2)	7 (35)	14 (23.8)	0.55
**Type of change** *									
1. Distress selling	50 (32.9)	28 (57.1)	8 (22.9)	14 (20.6)	–	–	–	–	
2. Selling at higher prices	–	–	–	–	47 (31.2)	36 (50)	5 (25)	6 (10.2)	
3. Reduced sales	38 (23.9)	10 (20.4)	11 (31.4)	17 (25.0)	11 (7.3)	2 (2.8)	2 (10)	7 (11.9)	
4. Increased sales	–	–	–	–	3 (2)	1 (1.4)	–	2 (3.4)	
**Change in farming schedule**	47 (30.9)	39 (79.6)	5 (14.3)	3 (4.4)	35 (23.2)	32 (44.4)	2 (10)	1 (1.7)	0.111
**Type of change**									
1. Early sowing	38 (25)	35 (71.5)	3 (8.6)	–	3 (2)	1 (1.4)	2 (10)	–	
2. Delayed sowing	8 (5.3)	3 (6.2)	2 (5.8)	3 (4.4)	1 (0.7)	–	–	1 (1.7)	
3. Not sowing	1 (0.7)	1 (2.1)	–	–	31 (20.6)	31 (43.1)	–	–	
**Reduced access to farm inputs**	74 (48.7)	6 (12.2)	21 (60)	47 (69.1)	35 (23.2)	–	7 (35)	28 (47.5)	0.223
Reasons*									
1. Inability to purchase seeds	60 (39.5)	6 (12.2)	19 (54.3)	35 (51.5)	21 (14)	–	5 (25)	16 (27.2)	
2. Inability to purchase fertilizer	51 (33.6)	–	12 (34.3)	39 (57.4)	32 (21.2)	–	6 (30)	26 (44.1)	
3. Inability to purchase seeds and fertilizers	43 (28.3)	–	10 (28.6)	33 (48.6)	19 (12.6)	–	4 (20)	15 (25.5)	
4. Reduced access to labor	22 (14.5)	–	6 (17.2)	16 (23.6)	7 (4.7)	–	1 (5)	6 (10.2)	
**Concern over future impact of COVID-19 on farming**									0.192
Ability to purchase seeds	72 (47.4)	7 (14.3)	20 (57.1)	45 (66.2)	33 (22.2)	–	6 (30)	27 (47.4)	
Ability to procure farm inputs	37 (24.3)	2 (4.1)	13 (37.1)	22 (32.3)	17 (11.5)	–	7 (35)	10 (17.6)	
Ability to sell crops	82 (53.9)	18 (36.7)	15 (42.9)	49 (72.1)	98 (65.8)	64 (88.9)	8 (40)	26 (45.7)	
Availability of manual labor	35 (23)	2 (4.1)	13 (37.1)	20 (29.4)	6 (4.1)	–	2 (10)	4 (7.1)	
**Changes in food access**									0.951
1. Easier	37 (24.4)	11 (22.4)	7 (20)	19 (27.9)	27 (17.9)	7 (9.7)	3 (15)	17 (28.8)	
2. Harder	54 (35.5)	12 (24.5)	13 (37.1)	29 (42.7)	22 (14.6)	(5.6)	6 (30)	12 (20.3)	
3. Same as before	61 (40.1)	26 (53.1)	15 (42.9)	20 (29.4)	102 (67.5)	61 (84.7)	11 (55)	30 (50.9)	
**Change in food prices**	136 (23.7)	45 (91.8)	28 (80)	63 (92.7)	137 (90.7)	68 (94.4)	12 (60)	57 (96.6)	0.659
**Change in sources of food access**	95 (62.5)	19 (38.8)	28 (80)	48 (70.6)	37 (24.5)	–	7 (35)	30 (50.8)	0.199
**Changes in diet**	66 (43.4)	37 (75.5)	17 (48.6)	12 (17.6)	33 (21.9)	17 (23.6)	4 (20)	12 (20.3)	0.866
**Reduced HH income**	117 (77)	42 (85.7)	28 (80)	47 (69.1)	64 (42.4)	31 (43.1)	7 (35)	26 (44.1)	0.307
**Reason for change** *									
1. Less opportunities for daily wage laboring	68 (44.7)	21 (42.9)	14 (40)	33 (48.5)	37 (24.6)	26 (36.2)	4 (20)	7 (11.9)	
2. Reduced sale of farm produce in local markets	54 (35.5)	27 (55.1)	15 (42.9)	12 (17.6)	25 (16.6)	8 (11.2)	4 (20)	13 (22.1)	
3. Others (reduced business, lack of transport, fear of infection, migration)	2 (1.3)	1 (2)	–	1 (1.5)	9 (6)	–	2 (10)	7 (11.9)	

† Chi-square test was used to determine the differences between lockdown (*N* = 152) and unlock (*N* = 151) phases.

* Multiple reasons were reported.

$ Negative impact of COVID-19 on food production include delayed/early cultivation, high prices of seeds and farm equipment, shortage of labor and manure, etc. whereas positive impact includes more production. HH, household.

## Data Availability

The raw data supporting the conclusions of this article will be made available by the authors, without undue reservation.
