# Supplementary material for: Effect of COVID-19 Pandemic on Food Systems and Determinants of Resilience in Indigenous Communities of Jharkhand State, India: A Serial Cross-Sectional Study
Source: Front Sustain Food Syst. Author manuscript; Available in PMC 2022 May 17. (PMC7612736; doi:10.3389/fsufs.2022.724321)
Supplement: Table S1 [file EMS144781-supplement-Table_S1.DOCX]

**Supplementary table 1: List of markets surveyed in Godda and Khunti districts of Jharkhand, India**

| **District** | **Market names** |
| --- | --- |
| **Godda** | *Agiamore Haat* |
|  | *Chandna Haat* |
|  | *Gariyal Chowk Haat* |
|  | *Imru Haat* |
|  | *Rampur Haat* |
|  | *Sindrimore Haat* |
| **Khunti** | *Torpa Haat* |
|  | *Tapkara Haat* |
