# Supplementary material for: Effect of COVID-19 Pandemic on Food Systems and Determinants of Resilience in Indigenous Communities of Jharkhand State, India: A Serial Cross-Sectional Study
Source: Front Sustain Food Syst. Author manuscript; Available in PMC 2022 May 17. (PMC7612736; doi:10.3389/fsufs.2022.724321)
Supplement: Table S2 [file EMS144781-supplement-Table_S2.DOCX]

**Supplementary table 2: Information on number of households approached and telephonically surveyed in Sauria Paharia, Santhal and Munda tribal communities of Jharkhand**

|  | **Total** | **Sauria Paharia**  **n** | **Santhal**  **n** | **Munda**  **n** |
| --- | --- | --- | --- | --- |
| **Lockdown phase** |  |  |  |  |
| Total HHs approached  *(Household list* + Snowball sampling)* | 1021  *(946 + 75)* | 292  (*250 + 42)* | 378  *(371 + 7)* | 351  *(325 + 26)* |
| Total HHs surveyed | 152 | 49 | 35 | 68 |
| Response rate (%) | 14.9% | 16.8% | 9.2% | 19.4% |
| **Unlock phase** |  |  |  |  |
| Total HHs approached  *(Household list* + Snowball sampling)* | 1088  *(946 + 142)* | 329  (*250 + 79)* | 380  *(371 + 9)* | 379  *(325 + 54)* |
| Total HHs surveyed | 151 | 72 | 20 | 59 |
| Response rate (%) | 13.8% | 21.8% | 5.3% | 15.6% |

*a list of tribal HHs with access to mobile phones or landline telephones obtained from the larger study.
